# DAF-16 and PQM-1: Partners in longevity

**DOI:** 10.18632/aging.100628

**Published:** 2014-01-06

**Authors:** Ronald G. Tepper, Coleen T. Murphy, Harmen J. Bussemaker

**Affiliations:** ^1^ Department of Biological Sciences, Columbia University, New York, NY 10027, USA; ^2^ Lewis-Sigler Institute for Integrative Genomics and Dept. of Molecular Biology, Princeton University, Princeton, NJ 08544, USA; ^3^ Center for Computational Biology and Bioinformatics, Columbia University Medical Center, New York, NY 10027, USA

Twenty years ago it was discovered that loss of insulin/IGF-1-like signaling (IIS) – such as occurs in *daf-2(−)* mutants – dramatically extends longevity in the nematode *C. elegans* via the FOXO transcription factor DAF-16 [1-3]. Under favorable conditions, DAF-16 remains cytosolic and transcriptionally inactive[2-4]; under stress, it is driven into the nucleus, leading to both up-regulation and down-regulation of large sets of genes, referred to as Class I and II, respectively [5]. Identifying these genes and their functions is key to understanding the molecular and biochemical determinants of aging and longevity. While several studies have been performed to determine the genes regulated by DAF-16, agreement on the identity of targets has been limited to a relatively small number of top responders [6]. Moreover, recent results have made it clear that while DAF-16 is responsible for the activation of Class I genes through the DAF-16 binding element (DBE), it does not interact directly with the upstream promoter regions of Class II genes, leaving the down-regulation of the latter in IIS mutants unexplained [7, 8].

To address these issues, we first performed a careful meta-analysis of all available genomewide expression profiles with DAF-16 active (nuclear) vs. inactive (cytosolic or null) [8]. We reprocessed relevant raw data from various laboratories, and used a voting algorithm developed specifically for this purpose to construct a consensus ranking of all *C. elegans* genes in terms of their responsiveness to DAF-16. This allowed us to redefine Class I and Class II targets with unprecedented sensitivity and specificity. Next, using a combination of computational and experimental methods, we discovered that the little-studied transcription factor PQM-1 regulates Class II genes (and Class I to a lesser extent), via the DAF-16 associated element (DAE), a GATA-containing motif previously lacking an identified binding factor [5]. Integrating our DAF-16 target ranking with ChIP-Seq data from the modENCODE project [9] showed that PQM-1 binding is strongly associated with both proximal upstream DAE occurrence and responsiveness to DAF-16. Indeed, a reporter gene assay confirmed that PQM-1 activates transcription in a DAE-dependent manner.

Next, we investigated whether and how PQM-1 subcellular localization depends on IIS status. Using GFP translational fusions, we found that the nuclear presence of PQM-1 and DAF-16 is controlled by IIS in opposite ways. A model emerged in which both the DBE and the DAE contribute to the expression of Class I genes, while Class II genes are exclusively controlled through the DAE. Under normal conditions, the DAE-dependent transcriptional activation of Class II genes by nuclear PQM-1 enables growth and development. Upon acute stress, PQM-1 leaves the nucleus while DAF-16 enters. The nuclear exit of PQM-1 causes expression of Class II genes to fall in response to loss of activation through the DAE; at the same time, DAF-16 moves into the nucleus, where its binding to the DBE in the upstream promoter region of Class I genes activates a stress response in the cell.

This model however was not yet complete: It was not obvious why the expression of Class II genes – which are only directly controlled by PQM-1 and not by DAF-16 – should rise when DAF-16 function is lost in a *daf-2*(−) background. To address this paradox, we performed additional experiments that revealed an active avoidance of DAF-16 and PQM-1 residing in the nucleus together. The translocation of DAF-16 to the nucleus in response to loss of DAF-2 signaling causes PQM-1 to be displaced to the cytoplasm; conversely, DAF-16 becomes more nuclear in wild-type (N2) worms after RNAi knockdown of PQM-1. Together, these observations suggest an elegant mechanism for switching between stress response and growth/development. While stress response is required for survival of an acute insult, it is likely to inhibit development and may be energetically costly to maintain. Through its antagonism with DAF-16, nuclear PQM-1 may help the worm maintain an “unstressed” transcriptional state that is critical to the animal's ability to develop. Loss of PQM-1 suppresses *daf-2* longevity and thermotolerance and further slows development. We also observed progressive loss of nuclear PQM-1 during wild-type aging, along with declining expression of its target genes. By day seven of adulthood, the tightly antagonistic coupling between DAF-16 and PQM-1 is lost, with both factors residing in the cytoplasm.

Despite our progress in elucidating the DAF-16 regulatory network, important questions remain unanswered. Does PQM-1 bind the DAE by itself, or as part of a complex with other factors? What molecular mechanisms govern PQM-1's subcellular distribution and its antagonistic interaction with DAF-16? How can loss of *pqm-1* in a *daf-2(−)* background, where PQM-1 is mostly cytosolic, cause a major reduction in lifespan? What mechanism underlies the nuclear exit of DAF-16 and PQM-1 with normal aging? Is loss of nuclear PQM-1 a cause or a consequence of aging? Is it a response to stress caused by unknown drivers of aging or is PQM-1 itself one of those drivers? Does PQM-1 have a functional equivalent in mammalian cells, as do many other components of the longevity pathway? Our discovery and initial characterization of PQM-1's important role in lifespan regulation provides a starting point for answering these questions.
